# Extensive Lateral Release and Medial Patellofemoral Ligament Reconstruction in 25 Years of Chronic Fixed Lateral Patellar Dislocation: A 5-Year Follow-Up Case Report

**DOI:** 10.1155/2019/9542398

**Published:** 2019-12-04

**Authors:** Andri M. T. Lubis, Petrus Aprianto, Yudistira P. Siregar

**Affiliations:** ^1^Department of Orthopaedics and Traumatology, Cipto Mangunkusumo General Hospital, Faculty of Medicine Universitas Indonesia, Jakarta, Indonesia; ^2^St. Carolus Bone and Joint Center, St. Carolus Hospital, Jakarta, Indonesia

## Abstract

Lateral dislocation of the patella is not uncommon and may impede daily activities as this causes compressive dysfunction and instabilities. Most cases of patellar lateral dislocation are due to damage to the medial patellofemoral ligament (MPFL), either rupture of detachment of the patella or femoral attachment. MPFL reconstruction alone was considered adequate for the treatment of this condition. We present a case of a 49-year-old male with chronic posttraumatic lateral patellar dislocation of the right knee of 25 years, which we treated with extensive lateral release and right medial patellofemoral ligament reconstruction with 5-year follow-up data.

## 1. Introduction

Patellofemoral disorders are one of the major complaints of all knee problems. This disorder may be caused by compressive dysfunction and less commonly by direct trauma [1]. Lateral patellar dislocation as one of the common patellofemoral disorders encountered by orthopaedic surgeons has the incidence of 23.2 per 100,000 persons per year. This number is higher than that previously reported by another study [2]. On the other hand, chronic fixed lateral patellar dislocation is uncommon, and until now, there is no data regarding its epidemiology. The anatomical abnormalities such as hypoplastic patella, deficient lateral femoral condyle, trochlear dysplasia, patella alta, excessive femoral anteversion, valgus deformity, and excessive *Q* angle could be associated with or become the predisposing factors of recurrent or habitual patellar dislocation [3].

The bony constraint of the femoral trochlea and the medial soft tissue structures keep the patella away from dislocating [4]. Furthermore, most imaging and surgical findings show that the medial patellofemoral ligament (MPFL) is the main soft tissue structure which stabilizes the patella against lateral dislocation [5]. It is responsible for 60% of the resistance to prevent lateral dislocation [6]. The MPFL lies in the anteromedial aspect of the knee and attaches between the adductor tubercle and the medial epicondyle of the femur. From its femoral insertion, the MPFL widens to an insertion in the half of the proximal medial patella area. The MPFL must either rupture or detach off the patella or femoral attachment site for dislocation to occur [7]. Kumar et al. [8] reported that MPFL reconstruction alone was adequate to treat lateral patellar instability as long as the tibial tubercle and trochlear groove (TT-TG) value was less than 20 mm and there was no trochlear dysplasia [9].

Many recent studies are focusing on the various techniques of MPFL repair, since it has so many variations. Surgical procedures can be divided into advancement or plication of tendons or ligaments, lateral retinacular release, osteotomy of the tibial tuberosity, trochleoplasty, purely soft tissue reconstruction, and combination of the above. The importance of surgical procedures is not only the technique that is used but also in the type of the graft. The semitendinosus, gracilis, quadriceps tendon, and synthetic grafts have been reported to be used, and there was no clear evidence about which one is superior [10].

This paper reported on the reconstruction of the MPFL after an extensive lateral release in a fixed lateral patellar dislocation. We explained the technique that was used to reduce and maintain the position of the patella and prevent it from redislocation. Besides, we also provided the 5-year follow-up data of using the semitendinosus tendon in MPFL reconstruction that had a similar result to the study by Smith et al. showing that the hamstring tendon graft was the preferred graft for MPFL reconstruction [11].

## 2. Case Presentation

The patient was a 49-year-old Caucasian male with body mass index of 27 kg/m^2^. He complained about pain on his right knee in the last 8 months. The pain was gradually increased and still could be relieved by rest. He had the history of fixed lateral patellar dislocation since 25 years before. The dislocation happened after he got slipped and his right knee hit the floor with the body on top/kneeling. He came into the examination room with antalgic gait without any walking aid. He felt that his right knee was giving away while walking. There was no other trauma. There were no history of medical and surgical treatment, no family with the same symptom, and no routine sport activity.

**Figure 1:**
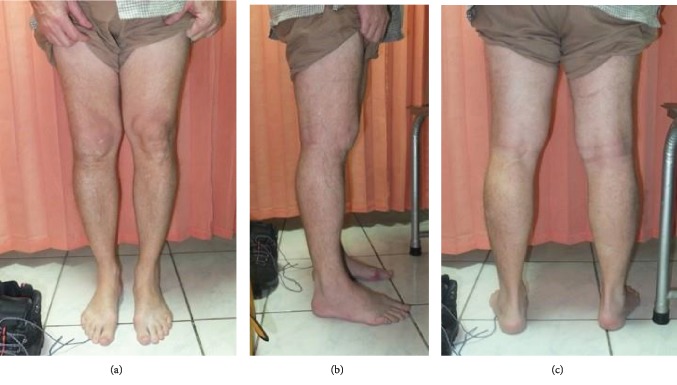
Atrophy muscle of the right thigh was seen from the anterior (a), lateral (b), and posterior (c) view.

Muscle atrophy could be seen on the anterior compartment of the thigh, specifically on the lateral medial and intermedius muscle. We observed the atrophy of the thigh muscle from the anterior, lateral, and posterior view (Figure 1). We found the patella was laterally dislocated. The knee range of motion was 0-120° in both passive and active movements. The patellar apprehension test was positive while the other special tests such as anterior and posterior drawer test, patellar tap test, joint line tenderness test, and Lachman test were all negative.

**Figure 2:**
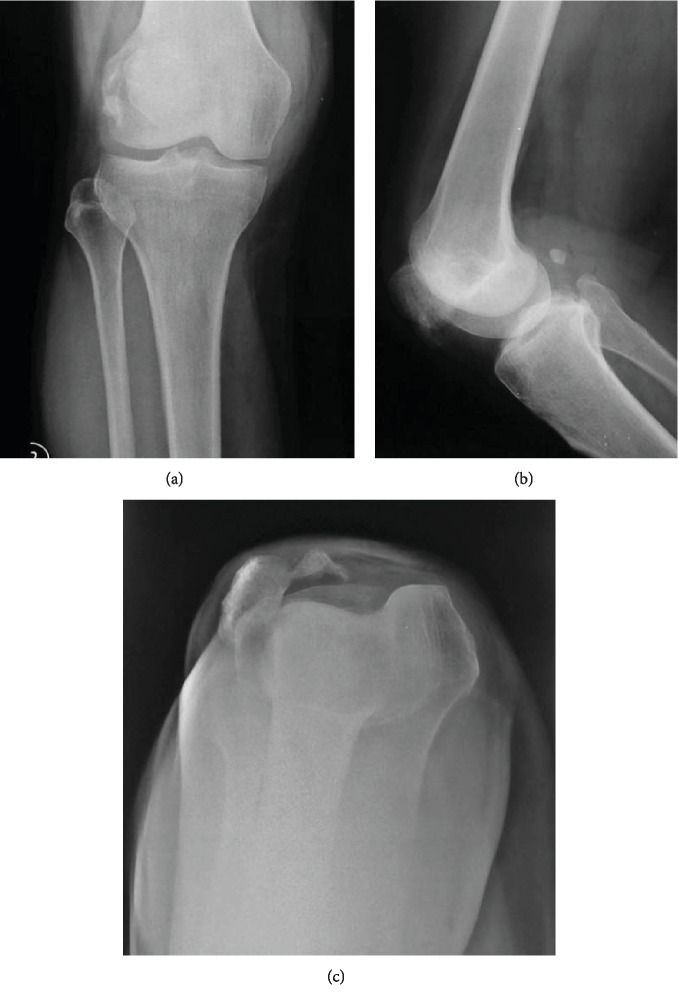
Radiographic imaging of the right knee in anteroposterior (a), lateral (b), and skyline (c) view showed lateral patellar dislocation.

On the AP view of the radiographic imaging, we found neither varus nor valgus deformity of the knee and no sclerosis at the subchondral bone. However, whole mechanical axis radiology was not possible to be performed at the time of examination; therefore, we are lacking data such as femoral anteversion and *Q* angle. There was no sign of secondary osteoarthritis of the knee joint. From the lateral view, there was no sign of patella alta and patella baja, but on the skyline view, we could see that there was a lateral dislocation of the knee. The trochlear groove was relatively normal (Figure 2). From the physical and X-ray examination, the patient was diagnosed as having chronically fixed lateral patella dislocation of the right knee.

**Figure 3:**
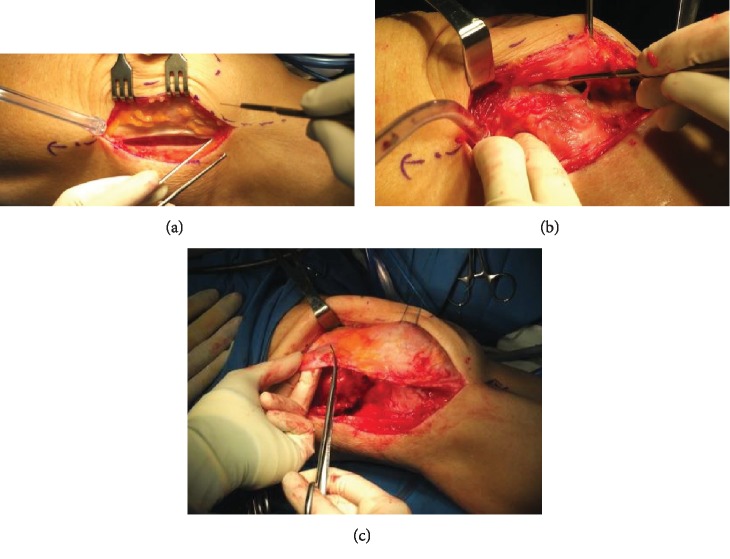
Extensive lateral release that included the fascia (a), lateral capsule (b), and vastus lateralis muscle (c).

We decided to perform extensive lateral release and right medial patellofemoral ligament reconstruction for this case. We performed incision on the lateral side of the knee joint until we reached the lateral capsule. We decided to release the lateral capsule and try to reduce the patella to be in the midline position. The patella was laterally fixed because of the tightened lateral vastus. We extended the incision and performed lateral release by cutting the fascia of the lateral vastus. After that, we tried again to reduce the patella but there was still resistance. We then cut the fascia of the medial vastus until the dislocated patella was fully released (Figure 3). After we successfully reduced the patella, we performed semitendinosus tendon graft harvesting.

A 3 cm oblique skin incision was made at the medial proximal tibial area, and the soft tissue was released. The semitendinosus tendon was harvested using a tendon stripper. We made a double-stranded graft by folding the semitendinosus tendon. The graft length was 160 mm, and the diameter of the ST tendon was 7 mm. After preparing the graft, 3 cm longitudinal skin incisions were made at the patellar and femoral natural footprints of the MPFL, and the soft tissue was peeled off so that the graft could pass through the soft tissue tunnel smoothly.

A patellar tunnel was made at the one-third of the superior part of the patella from the medial side until it reached the lateral side. Subsequently, the graft was passed through the bone tunnel of the patella. After the endobutton (Smith & Nephew, Andover, MA) reached the lateral side, it was flipped to fixate the graft end.

**Figure 4:**
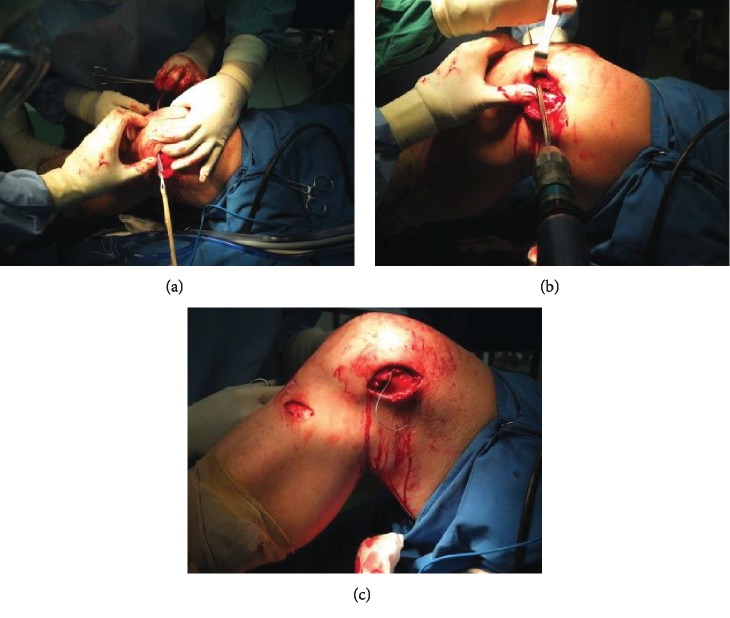
The step of MPFL reconstruction including the patellar side graft insertion (a). The drilling of the femoral part to make a tunnel at the Schottle point (b), and the insertion of the graft to the femoral side after the edge was sutured with ETHIBOND (c).

A femoral bone tunnel was constructed at the MPFL anatomical footprint, which is located at the Schottle point [12]. A 2.4 mm guide wire was inserted in the medial, ventral, and slightly proximal direction to avoid vessel and nerve injury. A 7 mm drill was used to create a bone tunnel. The depth of the bone tunnel was 80 mm until it reached the lateral cortex of the femur. We then sutured the graft end with ETHIBOND (Ethicon, Somerville, NJ) no. 2 and passed the graft through a medial side of the femur until the end of the tendon coming out from the lateral femoral side (Figure 4).

**Figure 5:**
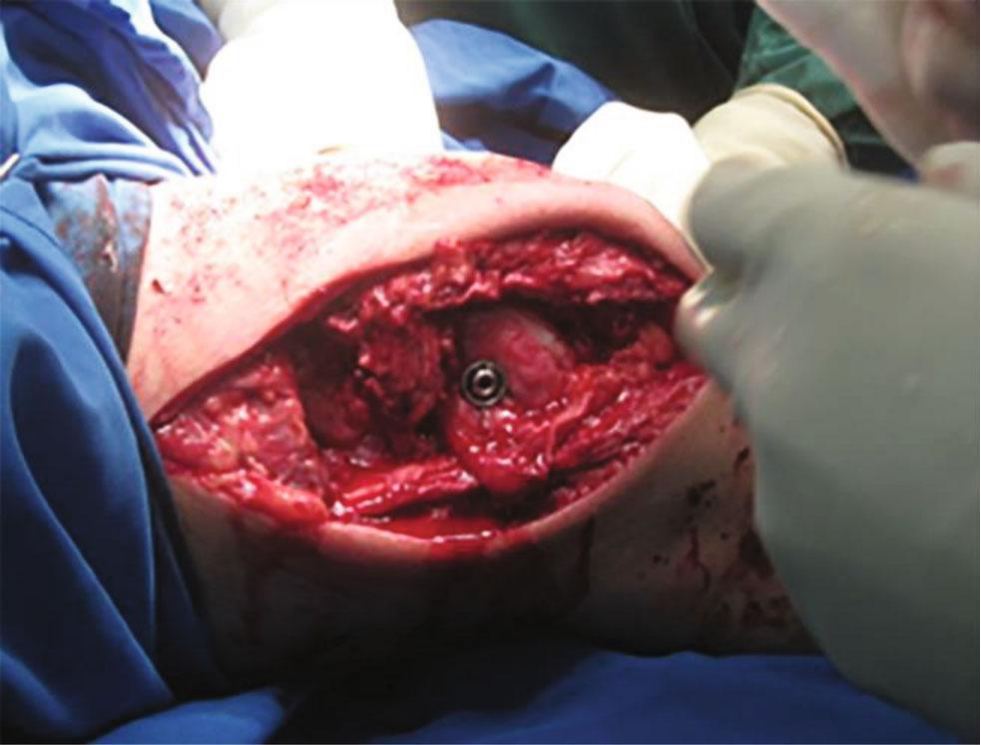
The cancellous screw and washer were put at the lateral side of the femur just above the femoral tunnel as the anchored point. The ETHIBOND suture was anchored to the screw and washer.

We then drilled the lateral side of the distal femur and put a cancellous screw and washer with a diameter of 4.5 and a length of 65 mm just above the femoral tunnel. The end of the graft was sutured with ETHIBOND, and the ETHIBOND suture was then anchored to the cancellous screw and washer as the fixation of the patella to prevent it from redislocation (Figure 5).

**Figure 6:**
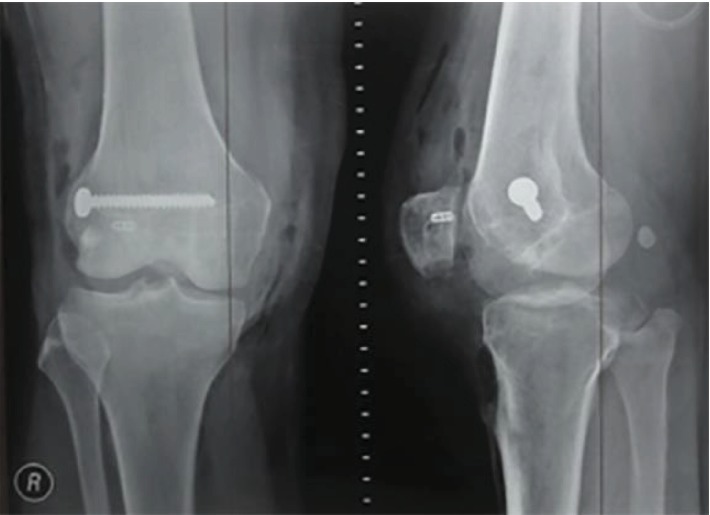
Radiographic examination to confirm the position of the endobutton, cancellous screw, and washer postoperatively.

The soft tissue was then closed in layers. After that, we did confirmation X-ray to confirm the position of the inserted screw at the lateral side of the distal femur (Figure 6). For the rehabilitation program, at first, the knee brace was applied to keep the knee in a full-extension position. The brace was used in this position up to three weeks; then, the knee flexion was increased to 20 degrees every week. After eight weeks postsurgery, the brace could be discharged and the patient could start active motion of the knee.

## 3. Discussion

Patellar dislocation could be caused by external factors such as trauma and internal factors such as skeletal and soft tissue abnormalities [13]. It could be divided into acute and chronic patellar dislocation. Chronic patellar dislocation could be found in many types of manifestations and must be differentiated with the habitual and obligatory patellar dislocation, which was also chronically manifested [14]. As the patella is constantly dislocated, the patella could not enter the trochlear groove, eventually causing pain, apprehension, and loss of function [15]. In our case, the patient was diagnosed with chronic fixed lateral patellar dislocation because the patella had been fixed in a dislocated position for 25 years after injury. We did not perform the MRI examination since the history, physical examination, and plain radiographic imaging showed patellar malposition laterally, which was the most probable cause of MPFL rupture. Radiographic examination revealed normal trochlea, contrary to trochlear dysplasia, which was prevalent in MPFL rupture cases.

In the chronic patellar dislocation patients, their muscles had changed in order to adapt with the dislocated patella. As in this case, we found muscle atrophy in the collateral side of the knee. Lateral vastus and medial vastus muscle tightness was found intraoperatively. These findings were also reported by Bistolfi et al. as conspicuous muscle wasting of each thigh on the medial side with depression on the medial vastus [16]. The muscle anatomical changes in this patient required that the initial lateral release of the tightened and fibrotic muscle must be done extensively so that the patella could be freed and reduced into its anatomical position. The lateral vastus and medial vastus insertion tendon were cut after the lateral capsule release had been proven to be inadequate for anatomical reduction of the patella.

Understanding the anatomy and characteristic of the MPFL is critical to perform a successful long-term MPFL reconstruction. Reconstruction of the medial patellofemoral ligament (MPFL) for recurrent lateral patellar dislocation is increasingly popular, and the use of a hamstring tendon graft for this purpose is accepted even though the fixation technique remained controversial [17]. Kumar et al. reported that the use of the gracilis tendon during MPFL reconstruction showed very satisfactory short-term results [9]. A systematic review by McNeilan et al. evaluated the ideal graft used in MPFL reconstruction. The use of the gracilis, semitendinosus, and quadriceps tendon autograft for a replacement of MPFL showed no significant differences based on its instability recurrence rate [18]. In this case, we used the semitendinosus tendon as a graft of choice to replace MPFL. The consideration of using this graft was because of the larger diameter of the semitendinosus compared to the gracilis tendon to hold the reduced patella. We considered that a bigger graft such as the semitendinosus played an important role for MPFL reconstruction, since the quadriceps tendon was out of choice in this case because the initial lateral release had involved this tendon.

Surgical techniques for MPFL reconstruction were varied in the matter of patellar and femoral fixation methods. Extensive release of the vastus muscles was obligatory as this is a chronic dislocation. It has been proven in this case that despite the extensive release, the outcome was satisfactory. There are the fixation of the ligament to the soft tissue like Chassaing's technique and many techniques that fixate the ligament to the bone such as the techniques performed by Schottle et al., Fink et al., and Camanho et al. [19]. So far, there is still no clear agreement about the best surgical technique among these techniques which have been showing good results. The position of the femoral tunnel also plays an important role. Most studies suggested the posterior aspect of the medial epicondyle, proximal to the medial collateral ligament and 1 cm distal to the adductor tubercle. Misplaced grafts will increase medial patellofemoral contact pressure [10].

As in this case, we used the Schottle point as the insertion point of the femoral part. This point was located 1.3 mm in front of a tangent to the posterior femoral cortex and 2.5 mm below the perpendicular line to this tangent passing by the point located at the junction between the posterior cortex and the lateral condyle [12]. This technique used an endobutton suture as the patellar fixation and the cancellous screw and washer through the bone tunnel as the femoral fixation. The cancellous screw was used because the insertion was held on the metaphyseal area of the distal femur. This is an unorthodox approach as common practice uses a biodegradable screw; however, this yields a satisfactory outcome and is more affordable. The graft was secured when the knee was flexed 30°.

For the patellar part graft fixation, some authors prefer a suture anchor technique to attach the graft into the patella. This procedure has a high failure rate without any known specific reason. A study by Arendt et al. has excluded possible causes of the failure including associated risk factors such as patella alta and trochlear dysplasia, abnormal tubercle-sulcus angle, excessive lateral tilt, or translation on axial radiographs, and none of the patients were operated on in the acute phase, when tissue inflammatory response might have aided in a more robust healing response. After doing so, the failure rate in the study was still high [5]. In this case, we drilled the patella on the superior one-third region to prevent the fracture of the patellar bone and on 60° of knee flexion. Some surgeons suggest not to drill the patella due to the risk of patellar fracture. However, this risk can be avoided by using a small (5 mm in diameter) and short (20 to 25 mm in length) tunnel. By using this technique, patellar fracture can be avoided [20].

We fix the graft using endobutton instead of a screw to minimize the risk of patellar fracture. It was supported by the study that has been done by Lorbach et al. that demonstrated that despite the tunnel location, the angle of knee flexion to MPFL graft fixation should be concerned to avoid overtension of the graft. Lorbach et al. show that the optimal angle of knee flexion is 60°, which was also performed in this patient. The lower flexion angles may lead patellofemoral cartilage to medial overload causing stiffness, subsequent osteoarthritis, and pain [21].

Complications of MPFL reconstruction are commonly recurrent dislocation, patellar fracture, improper anatomic placement of the graft, and overtightening, leading to stiffness and pain. When the drilling is performed, the patellar fracture could happen because of thermal damage. Therefore, we performed the drilling few millimeters at a time and sequentially clean the drill to remove bony debris. By using this technique, patellar fracture can be avoided [20].

**Figure 7:**
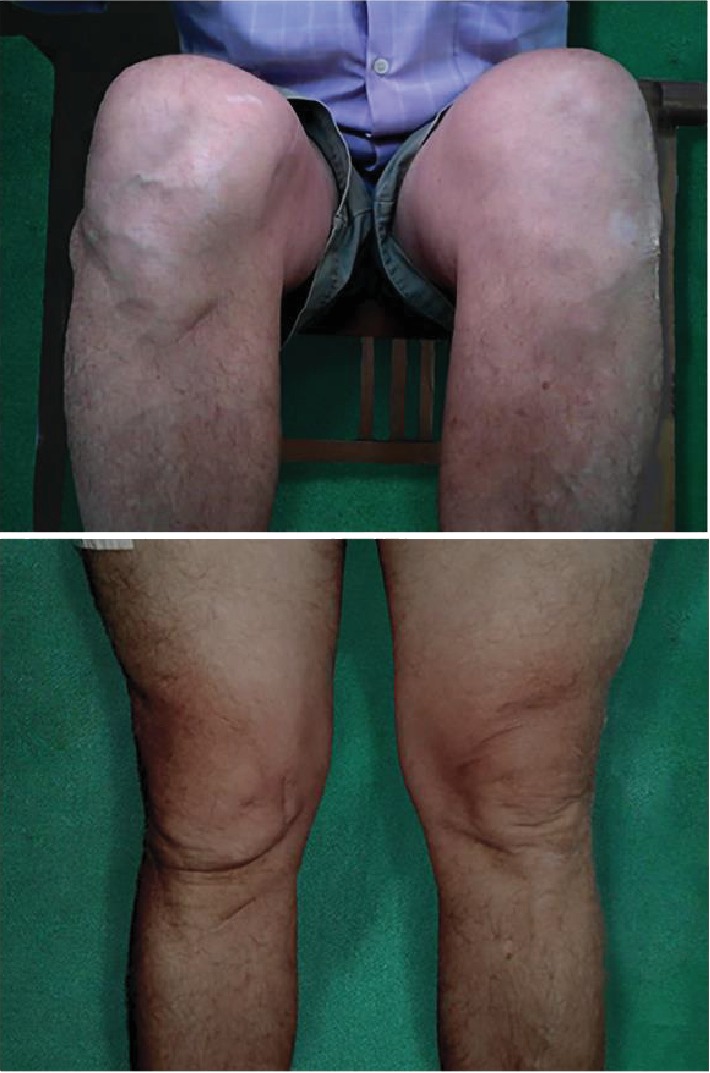
A 5-year follow-up showed no redislocation of the right knee in flexion and extension.

In our case, we did a 5-year follow-up of the patient. The patient did not complain about any pain, instability, and the feeling that the knee was giving away. The patient could stand and walk normally without any walking aids and could sit with flexed knee without any complaint (Figure 7). Another complication that could be happened was sensory deficit of the donor graft area, but it also did not happen in our case [22].

Chronic fixed lateral patellar dislocation is a rare case that must be treated differently than other chronic types of patellar dislocation. An extensive lateral release should be done to release the patella from the surrounding muscles and fibrotic tissues. We performed a modified MPFL reconstruction technique with a screw and washer as an anchor at the femoral side. The use of endobutton on the patellar side showed an excellent outcome. The use of the semitendinosus muscle tendon as a graft had been proven to be an ideal graft as a replacement of MPFL. There were no redislocation, pain, and stiffness found during five years of follow-up.
